# Anterior Gradient 2 is a Significant Prognostic Biomarker in Bone Metastasis of Breast Cancer

**DOI:** 10.3389/pore.2022.1610538

**Published:** 2022-11-03

**Authors:** Jin-Jin Li, Shuai Wang, Zhong-Ning Guan, Jin-Xi Zhang, Ri-Xin Zhan, Jian-Long Zhu

**Affiliations:** ^1^ Department of Orthopaedics, Hangzhou Ninth People’s Hospital, Hangzhou, China; ^2^ Department of Pathology, Hangzhou Ninth People’s Hospital, Hangzhou, China; ^3^ Department of Medical Record Management, Hangzhou Ninth People’s Hospital, Hangzhou, China

**Keywords:** prognosis, bone metastasis, DEGs, BRCA, AGR2

## Abstract

**Background:** The study aimed to detect DEGs associated with BRCA bone metastasis, filter prognosis biomarkers, and explore possible pathways.

**Methods:** GSE175692 dataset was used to detect DEGs between BRCA bone metastatic cases and non-bone metastatic cases, followed by the construction of a PPI network among DEGs. The main module among the PPI network was then determined and pathway analysis on genes within the module was performed. Through performing Cox regression, Kaplan-Meier, nomogram, and ROC curve analyses using GSE175692 and GSE124647 datasets at the same time, the most significant prognostic biomarker was gradually filtered. Finally, important pathways associated with prognostic biomarkers were explored by GSEA analysis.

**Results:** The 74 DEGs were detected between bone metastasis and non-bone metastasis groups. A total of 15 nodes were included in the main module among the whole PPI network and they mainly correlated with the IL-17 signaling pathway. We then performed Cox analysis on 15 genes using two datasets and only enrolled the genes with *p* < 0.05 in Cox analysis into the further analyses. Kaplan-Meier analyses using two datasets showed that the common biomarker AGR2 expression was related to the survival time of BRCA metastatic cases. Further, the nomogram determined the greatest contribution of AGR2 on the survival probability and the ROC curve revealed its optimal prognostic performance. More importantly, high expression of AGR2 prolonged the survival time of BRCA bone metastatic patients. These results all suggested the importance of AGR2 in metastatic BRCA. Finally, we performed the GSEA analysis and found that AGR2 was negatively related to IL-17 and NF-kβ signaling pathways.

**Conclusion:** AGR2 was finally determined as the most important prognostic biomarker in BRCA bone metastasis, and it may play a vital role in cancer progression by regulating IL-17 and NF-kB signaling pathways.

## Introduction

Breast cancer (BRCA) has become one of the deadliest types of tumors in women worldwide (1). With the progress of diagnosis and treatment technology, primary BRCA no longer poses a serious threat to patients’ lives; instead, metastasis acts as the foremost cause of death (2). At present, distant metastasis has become the primary cause of cancer-related death and occurs in about 20%–30% of BRCA patients (3). It has been noted that BRCA showed a proclivity to metastasize to specific organs, such as the lungs and bones (4). Bone is one of the most common sites of distant breast cancer metastasis (5). Normally, the rigid bone matrix can be able to restrict the growth of tumor cells; however, bone is also a rich reservoir of nutrients and growth factors to support tumor growth through the vicious cycle of osteolytic metastasis (6). Targeting osteoclasts might be a promising approach to stopping bone metastasis. A previous study by Coleman et al. showed that more than 65% of advanced-stage BRCA patients developed bone metastasis (7). The occurrence of bone metastasis exerts a dramatic impact on a patient’s quality of life and causes significant pain, physical disability, and potential loss of employment (8). At present, some therapeutic drugs including bisphosphonates, anti-RANKL antibodies, and Denosumab have been applied as osteoclast targeting agents to inhibit bone metastasis. However, the clinical benefit of these therapeutic agents is unsatisfactory due to their side effects, high cost, or minimal long-term benefit (9). The median overall survival of patients with BRCA metastasis remains unfavorable (10). To further improve disease outcomes, there is an urgent need to explore the biomarkers associated with metastasis in breast cancer.

BRCA metastasis is a complex biological process, in which multiple genes interact and influence each other (11). A range of genes presents the regulation effects on tumor cells, including genes that promote or inhibit metastasis. Screening metastasis-related genes can provide us clues for studying tumor metastasis targets and understanding the potential pathogenic mechanism. Wang et al. constructed a new and convenient prediction model based on 6 genes for predicting the lung metastasis risk of clinical BRCA patients (12). Lu et al. suggested that 5 hub genes were associated with human epidermal growth factor receptor 2 (HER2) positive BRCA with brain metastasis, and the ribosomal pathway seemed to play a very important role (13). Tang et al. determined that 4 crucial genes were associated with the overall survival of BRCA patients, which correlated with BRCA brain metastasis (14). Regarding bone metastasis, Zhang et al. concluded that four genes may play a central role in BRCA bone metastasis (15). Li et al. indicated that CST6 protein and peptides inhibited the BRCA bone metastasis by suppressing CTSB activity and osteoclastogenesis (6). Spadazzi C et al. showed that Trefoil factor-1 (TFF1) upregulation in estrogen-receptor (ER) positive BRCA correlated with an increased risk of bone metastasis (16). Haider et al. suggested that interleukins were the mediators of the tumor cell-bone cell crosstalk during the initiation of BRCA bone metastasis, and interleukins showed potential as therapeutic targets in preventing metastatic outgrowth in bone (17). The expression pattern of these genes associated with metastasis might determine the potential for tumor cell metastasis (18). It was necessary to reveal more differentially expressed genes involved in BRCA bone metastasis.

In the present study, we performed a bioinformatics analysis to explore the BRCA bone metastasis-related biomarkers. The dataset of GSE175692 was obtained to discriminate differentially expressed genes (DEGs) between bone metastatic cases and non-bone metastatic cases. Furthermore, the GSE175692 and GSE124647 datasets were used to filter prognosis-associated genes in BRCA bone metastatic samples. Finally, we performed the gene set enrichment analysis (GSEA) to reveal the significant pathways associated with the prognostic biomarker.

## Methods

### Identification of DEGs

In this study, we obtained the gene expression profile of the GSE175692 dataset from the Gene Expression Omnibus (GEO) database. The Robust Multi-array Average (RMA) method was used for pre-processing, including background correcting, normalizing, and calculating expression. The dataset containing 31 BRCA bone metastatic samples and 149 non-bone metastatic samples was used to detect the differentially expressed genes (DEGs) associated with BRCA bone metastasis. Subsequently, the Limma package in R was then used to screen the DEGs. The inclusion criteria for the DEGs were that upregulated genes must have a log2 fold change (logFC) ≥ 2 and an adjusted *p*-value <0.05, while downregulated genes must have a logFC < −2 and an adjusted *p*-value < 0.05. The volcano map was used to visualize the DEGs, and a bubble plot was used to present ranked DEGs.

### PPI Network Construction and Main Module Analysis

We then used the String database to construct the protein-protein interaction (PPI) network among all DEGs. The organism was *Homo sapiens*, and the minimum required interaction score was medium confidence. Then Cytoscape was used to visualize the PPI network. Among the whole PPI network, the most significant module containing 15 nodes was identified by MCODE analysis in Cytoscape. The 15 nodes within the module were considered the hub genes. Regarding the 15 genes, we used the R package of clusterProfile to perform Gene Ontology (GO) and Kyoto Encyclopedia of Genes and Genomes (KEGG) enrichment analysis, and the top 5 terms were presented. The GO terms associated with module genes were annotated from aspects of biological process, cellular component, and molecular function. *p* < 0.05 was considered statistically significant.

### Filtration of Prognosis-Associated Biomarkers in BRCA Metastasis

Regarding the 15 hub genes within the main module, univariate Cox analysis was performed to initially explore the prognosis-related biomarkers. The GSE175692 (*N* = 180) and GSE124647 (*N* = 140) datasets, all containing patients with BRCA metastasis, were separately used to evaluate the correlation between 15 gene expression levels and overall survival time by univariate Cox regression analysis. The clinicopathologic parameters of the patients with BRCA metastasis in GSE175692 and GSE124647 were presented in Table 1.

**TABLE 1:** Clinical pathological parameters of patients with breast cancer metastasis in the GEO cohort.

	Characteristics	Subgroup	Number	Percent
GSE175692				
	Age (years)	Percentiles	(25, 50, 75)	(16, 32, 54)
	Overall survival time (months)	Percentiles	(25, 50, 75)	(49, 57, 70)
	Overall survival status	Alive	76	42.2%
		Dead	104	57.8%
	Gender	Male	1	0.6%
		Female	179	99.4%
	Pre-post menopausal	Premenopausal	68	37.8%
		Postmenopausal	102	56.7%
		Unknown	10	5.5%
	Relapsed or *de novo* metastasis	Relapsed	148	82.2%
		*De Novo*	29	16.1%
		Unknown	3	1.7%
	Site of metastatic biopsy	Distant	130	72.2%
		Locoregional	50	27.8%
	Metastatic spread	Bone	31	17.2%
		Brain	22	12.2%
		Breast	24	13.3%
		Liver	30	16.7%
		Lung	12	6.7%
		Lymph Node	16	8.9%
		Muscle	2	1.1%
		Ovary	4	2.2%
		Peritoneum	2	1.1%
		Pleura	10	5.6%
		Skin	27	15.0%
	metastatic spread: visceral	Yes	148	82.2%
		No	31	17.2%
		Unknown	1	0.6%
	IHC group	Her2+	17	9.4%
		Hr+/Her2-	98	54.4%
		Tnbc	51	28.3%
		Unknown	14	7.8%
GSE124647				
	Overall survival time (months)	Percentiles	(25, 50, 75)	(12, 24, 41)
	Overall survival status	Alive	43	30.7%
		Dead	97	69.3%
	Progression-free survival (months)	Percentiles	(25, 50, 75)	(2.5, 5.5, 14.3)
	Progression-free survival status	Free	10	7.1%
		Progressed	130	92.9%
	PR status	Positive	80	57.1%
		Negative	60	42.9%
	Visceral disease	Yes	80	57.1%
		No	60	42.9%
	Prior endocrine sensitivity	Yes	70	50.0%
		No	39	27.9%
		Unknown	31	22.1%
	Stage IV at initial diagnosis	Yes	45	32.1%
		No	95	67.9%

Abbreviations: GEO, Gene Expression Omnibus database; PR status, progesterone receptor status; IHC, Immunohistochemistry; HER2, human epidermal growth factor receptor 2; HR, hormone receptor; TNBC, triple-negative breast cancer.

Subsequently, the correlation between gene expression value and overall survival was confirmed by performing Kaplan-Meier analysis using GSE175692 and GSE124647 datasets, respectively. Only the genes with *p* < 0.05 in Cox analysis were selected for the Kaplan-Meier analysis. Before Kaplan-Meier analysis, all patients were divided into low and high expression groups according to the best cut-off value of gene expression level. In order to determine the best cut-off value, all possible cutoff values between the lower and upper quartiles were computed, and the best performing threshold was used as the cutoff. The survival difference between the two groups was compared by a log-rank test. *p* < 0.05 was considered the statistical significance.

To further determine the significance of prognosis-related hub genes, we constructed a prognostic nomogram and receiver operating characteristic (ROC) curve analysis using the data of these two datasets as well. Only the genes with *p* < 0.05 in Cox analysis were selected for nomograms and ROC analysis. Through nomograms, the detailed contribution of hub genes to survival probability can be determined. In addition, the prognostic performance of hub genes on the survival of patients was also investigated by ROC curve analysis, and the area under the curve (AUC) was set as an evaluation indicator. *p* < 0.05 was considered statistically significant.

### The Detailed Role of AGR2 in BRCA Bone Metastasis and Non-Bone Metastasis

After a series of analyses, our study finally determined that AGR2 was the most vital biomarker among all hub genes. We subsequently used the GSE175692 dataset to detect its differential expression in BRCA bone metastatic cases and non-bone metastatic cases. The Student’s t-test was used to compare the AGR2 expression level between the two groups. The prognostic impact of AGR2 on the overall survival in BRCA bone metastatic cases and non-bone metastatic cases was then explored using Kaplan-Meier analysis, followed by ROC analysis. Before survival analysis, all the samples were divided into high and low expression groups according to the best cut-off value of the AGR2 expression level. The survival difference between high and low expression groups was compared with the log-rank test. *p* < 0.05 was considered statistically significant.

### The Clinical Value of AGR2 in Primary Breast Tumor

Further, we evaluated the clinical value of AGR2 in primary breast tumors. The clinical data of BRCA patients and the expression value of AGR2 were obtained from the TCGA database. The patients without AGR2 expression value and survival information were excluded. The clinical pathological parameters of patients were presented in Table 2. Using the TCGA-BRCA dataset, we assessed the expression difference of AGR2 regarding the molecular subtypes and nodal status. In addition, we also performed survival analysis on AGR2 regarding different molecular subtypes using the Kaplan-Meier method and log-rank test. Before survival analysis, all the samples were divided into high and low expression groups according to the best cut-off value of the AGR2 expression level. *p* < 0.05 was considered statistically significant.

**TABLE 2:** Clinical pathological parameters of patients with breast cancer in the TCGA-BRCA cohort.

Characteristics	Subgroup	Number	Percent
Age (years)	Percentiles	(25, 50, 75)	(49, 59, 68)
Overall survival time (days)	Percentiles	(25, 50, 75)	(441, 822, 1,649)
Gender	Male	12	1.1%
	Female	1,063	98.9%
Cancer stage	I	181	16.8%
	II	619	57.6%
	III	246	22.9%
	IV	20	1.9%
	X	9	0.8%
ER status	Positive	795	74.0%
	Negative	231	21.5%
	Unknown	49	4.6%
PR status	Positive	686	63.8%
	Negative	337	31.3%
	Unknown	52	4.8%
HER2 status	Positive	163	15.2%
	Negative	551	51.3%
	Unknown	361	33.6%

Abbreviations: TCGA, The Cancer Genome Atlas; BRCA, breast cancer; ER, estrogen receptor; PR, progesterone receptor; HER2, human epidermal growth factor receptor 2.

### GSEA Analysis on AGR2

The KEGG analysis has indicated the top five pathways associated with hub genes. We further performed the gene set enrichment analysis (GSEA) to reveal the correlation between AGR2 and these five pathways in BRCA metastatic samples. The expression profile for GSEA analysis was from the GSE124647 dataset. In addition, we also explored the correlation between AGR2 and three other important metastasis-related pathways, including nuclear factor kappa B (NF-kB), tumor necrosis factor-beta (TNF-beta), and mammalian target of rapamycin (mTOR) signaling pathways. For GSEA analysis, the criterion for permutations number was 1,000, enrichment statistic was weighted, and the metric for ranking genes was Pearson.

## Results

### DEGs Identification and Enrichment Analysis

**FIGURE 1:**
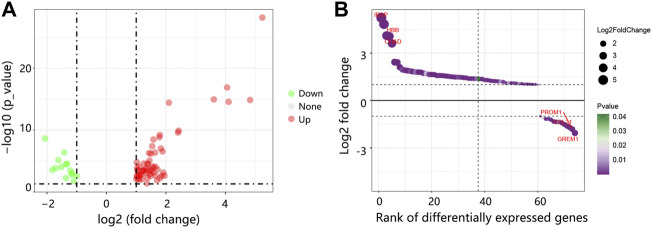
The DEGs identification between BRCA bone metastasis and non-bone metastasis groups in GSE175692. **(A)** Volcano map of DEGs. Red: upregulated genes. Green: downregulated genes. **(B)** Bubble plot of the ranked DEGs. Abbreviation: DEGs, differentially expressed genes; BRCA, breast cancer.

Through GSE175692, we detected 74 DEGs between bone metastasis and non-bone metastasis groups, containing 60 upregulated DEGs and 14 down-regulated DEGs (Figure 1A). The top three upregulated and downregulated DEGs can be found in Figure 1B.

**FIGURE 2:**
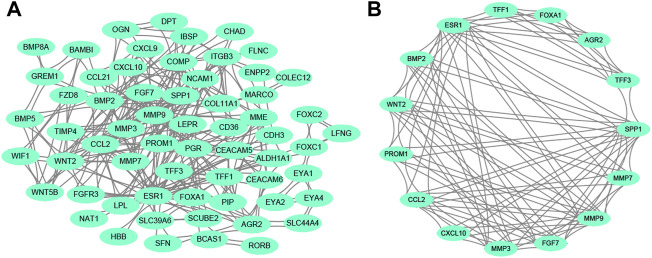
PPI network analysis. **(A)** The whole PPI network among DEGs. **(B)** The main module in the whole PPI network is detected by MCODE analysis. Abbreviation: PPI, protein-protein interaction. DEGs, differentially expressed genes.

Regarding all the DEGs, a PPI network was constructed. The PPI network contained 65 nodes and 354 edges (Figure 2A). From the whole network, the most significant module with the highest score was determined by MCODE analysis. The core module contained 15 nodes and 104 edges (Figure 2B), and these 15 nodes were regarded as the hub genes. Our result suggested that the module and 15 genes might play an important regulatory role in the whole network.

**FIGURE 3:**
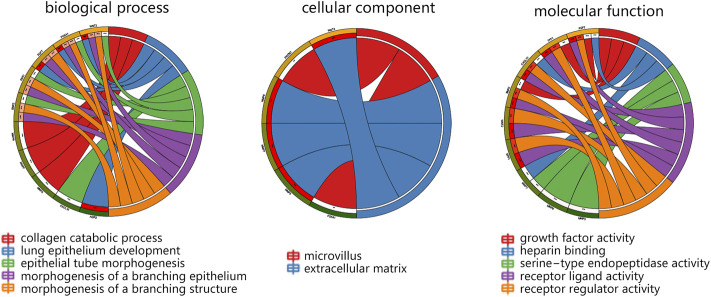
The GO analysis on 15 hub genes within the main module. Abbreviation: GO, Gene Ontology.

In terms of 15 hub genes, the vital GO terms were annotated from aspects of biological process, cellular component, and molecular function. The analysis in Figure 3 showed that 15 genes mainly participated in the collagen catabolic process, and were located in the extracellular matrix. For molecular function, 15 hub genes were mainly involved in growth factor activity and receptor activity.

**FIGURE 4:**
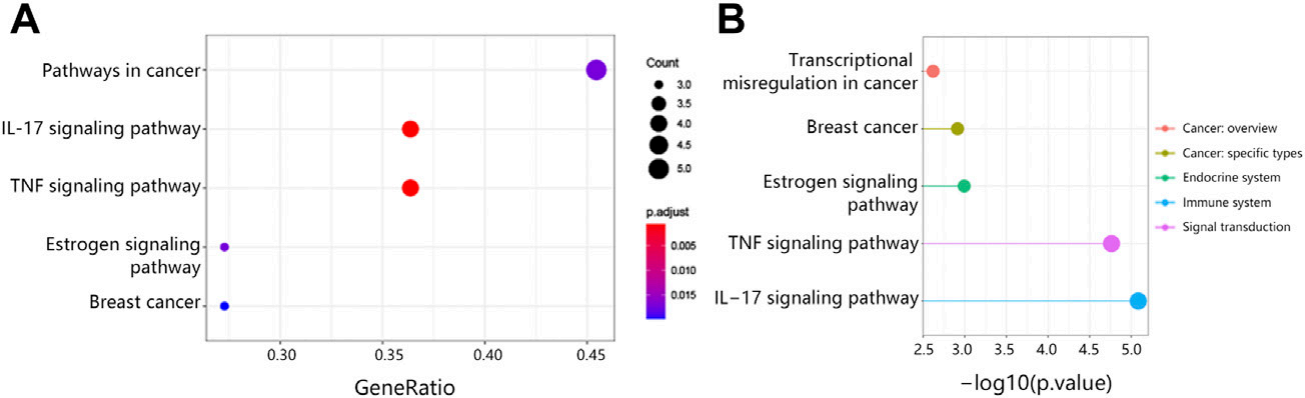
The KEGG pathway analysis associated with 15 hub genes. **(A)** The top5 KEGG pathways. **(B)** Pathway classification. Abbreviation: KEGG, Kyoto Encyclopedia of Genes and Genomes.

The study also explored the possible pathways associated with 15 hub genes *via* KEGG analysis. We found that 15 hub genes significantly correlated with the cancer pathway, IL-17 signaling pathway, TNF signaling pathway, breast cancer, and estrogen signaling pathway (Figure 4A). It followed that these pathways were associated with the endocrine system, immune system, and signal transduction (Figure 4B).

### Significant Hub Genes Identification Associated With Patient’s Prognosis

Subsequently, the GSE175692 and GSE124647 datasets were separately used to detect the most significant prognosis-associated hub genes among 15 genes. The univariate Cox regression analysis was initially performed to filter the hub genes, and only the genes with *p* < 0.05 in Cox analysis were enrolled into the further Kaplan-Meier analysis, nomogram, and ROC analysis. Combining the Cox, Kaplan-Meier, nomogram, and ROC analyses, the most important prognosis-related genes can be gradually determined.

**FIGURE 5:**
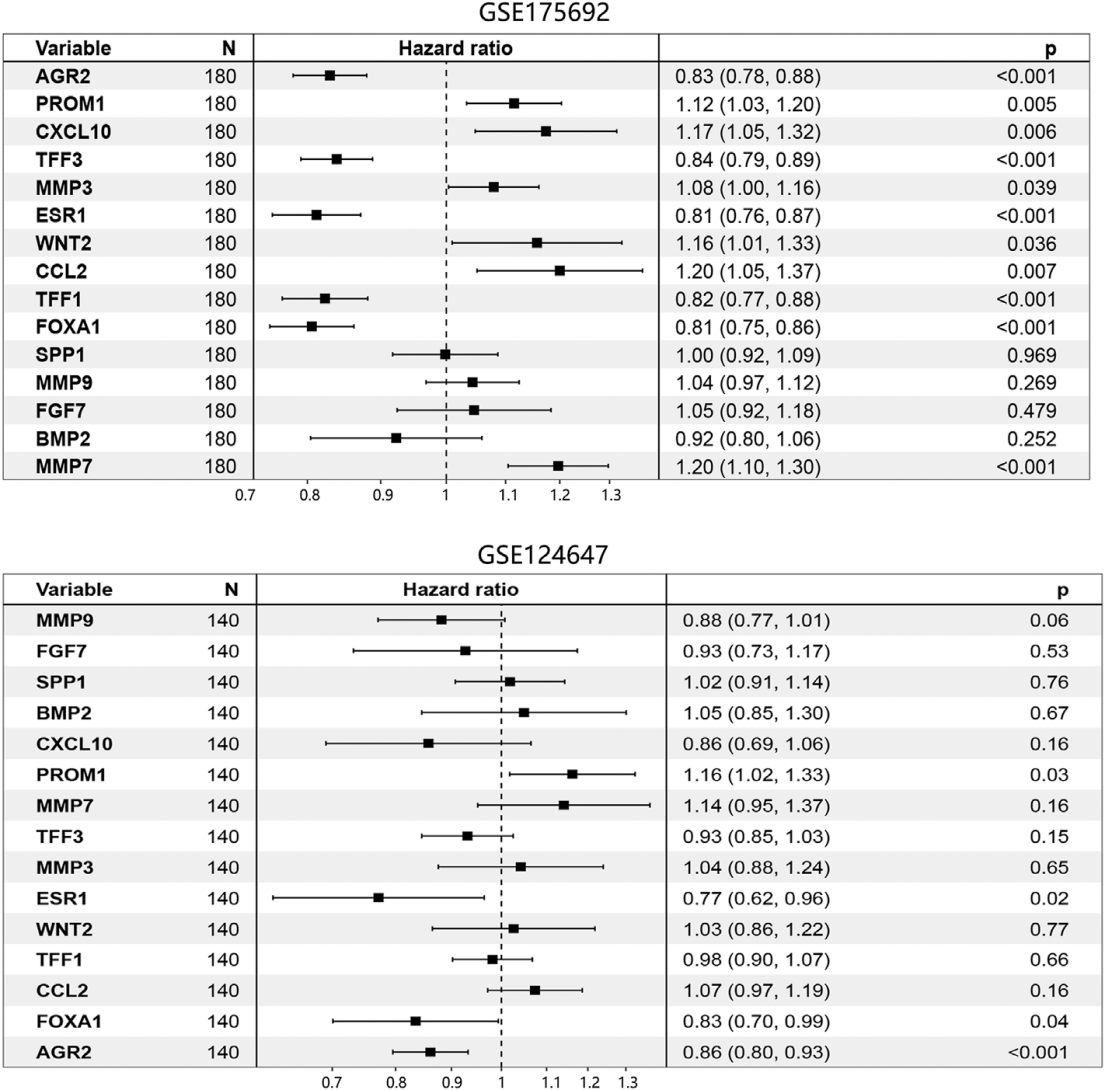
Prognosis-associated hub genes determination by Cox regression analysis in BRCA metastasis using GSE175692 and GSE124647 datasets. The number in brackets was 95% CI of the hazard ratio. Abbreviation: N, sample number; CI, confidence interval; BRCA, breast cancer.

**FIGURE 6:**
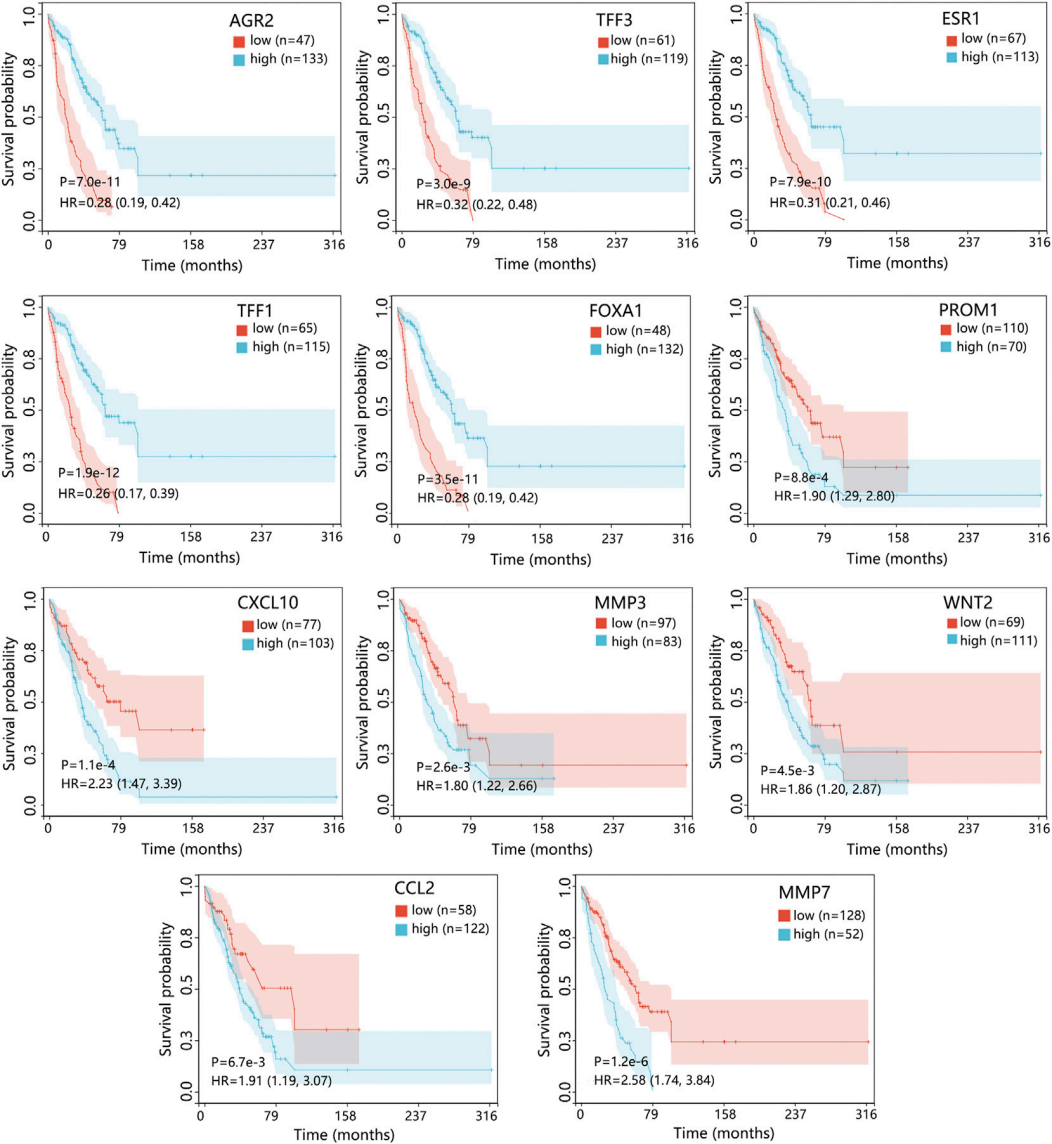
The prognostic value of 11 hub genes in patients with BRCA metastasis using the GSE175692 dataset. The genes with *p* < 0.05 in Cox analysis were selected for Kaplan-Meier analysis. The comparison result was presented with *p* value and HR (95% CI). Abbreviation: HR, hazard ratio; CI, confidence interval; BRCA, breast cancer.

Cox regression analysis using the GSE175692 dataset (Figure 5) initially indicated that 11 hub genes presented significant prognostic impact (all *p* < 0.05). The Kaplan-Meier analysis further confirmed the correlation between the expression value of these 11 genes and the overall survival time of patients with BRCA metastasis (Figure 6). Taken together, our results indicated that high expressions of AGR2, TFF3, ESR1, TFF1, and FOXA1 were related to longer overall survival time of patients (all *p* < 0.05). However, high expressions of PROM1, CXCL10, MMP3, WNT2, CCL2, and MMP7 were related to the poor prognosis of patients (all *p* < 0.05).

**FIGURE 7:**
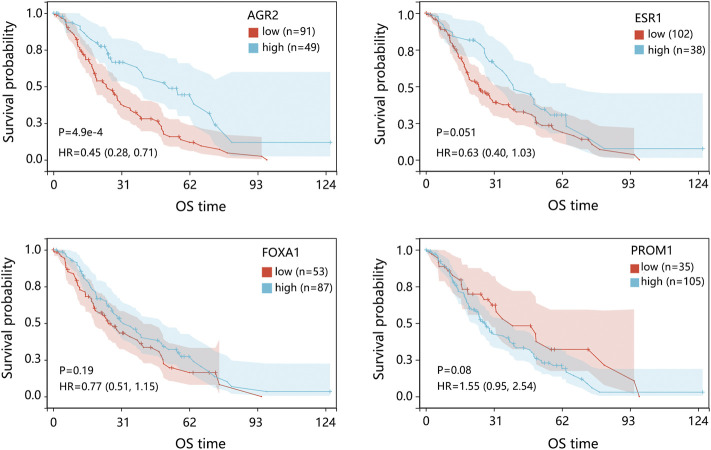
The prognostic impact of 4 hub genes in patients with BRCA metastasis using the GSE124647 dataset. The genes with *p* < 0.05 in Cox analysis were selected for Kaplan-Meier analysis. The comparison result was presented with *p* value and HR (95% CI). Abbreviation: HR, hazard ratio; CI, confidence interval; BRCA, breast cancer.

The Cox regression analysis using the GSE124647 dataset showed that only 4 genes were related to the patient’s prognosis (Figure 5). Kaplan-Meier survival analysis then indicated that only AGR2 expression level was associated with the overall survival of patients. Compared with the low expression group, the AGR2 high expression group showed a longer survival time (Figure 7, *p* = 0.0044).

**FIGURE 8:**
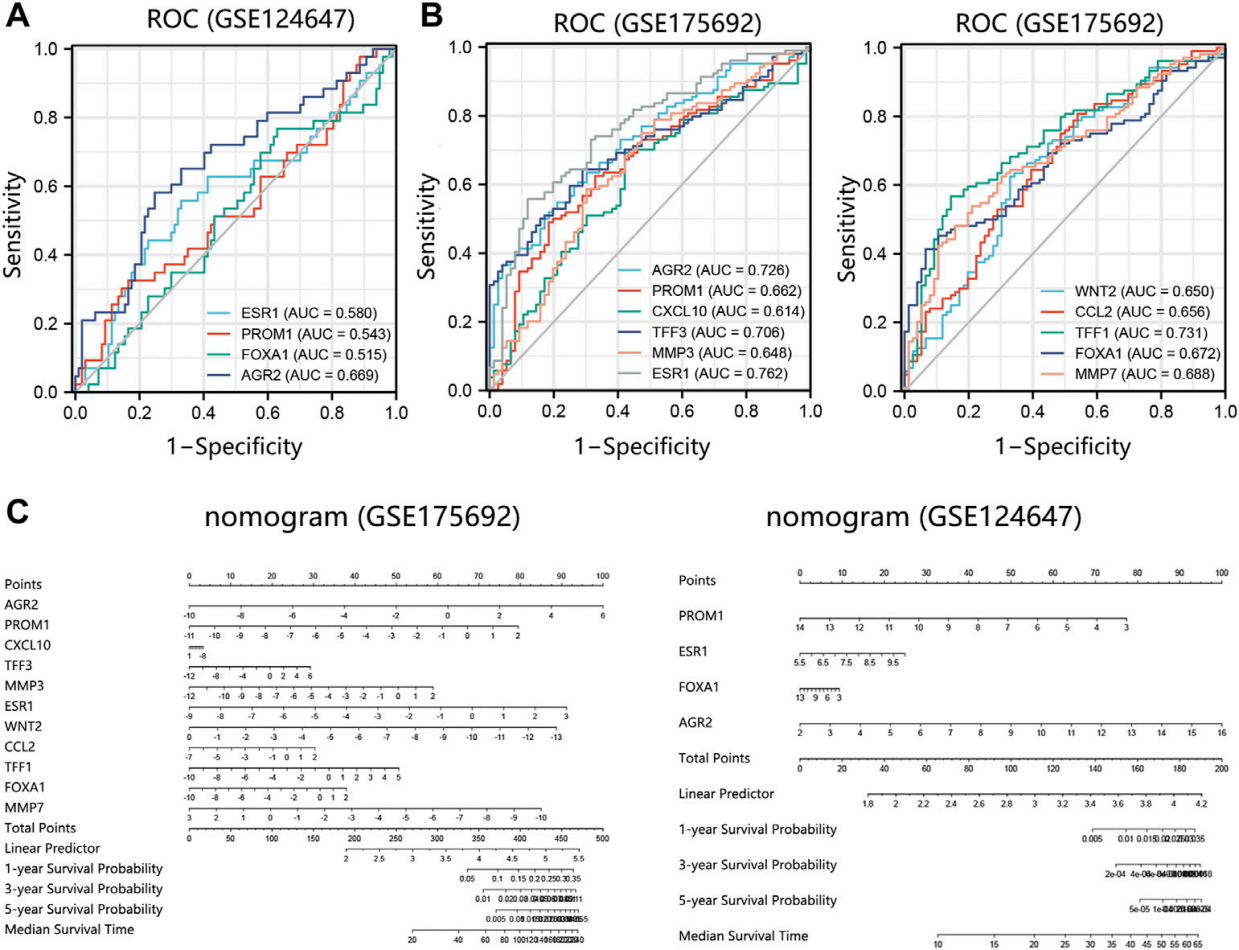
Prognostic performance analysis on prognosis-related hub genes in 2 datasets. **(A)** ROC analysis on 4 hub genes in the GSE124647 dataset. **(B)** ROC analysis on 11 hub genes in the GSE175692 dataset. **(C)** Nomogram analysis. Points are the scores corresponding to a single gene. Total points are the sum of the Points that all genes. The length of the line corresponding to each gene in the prognostic nomogram reflects the contribution of each gene to one patient’s outcome. The hub genes with *p* < 0.05 in Cox regression analysis were enrolled into the nomogram and ROC analysis. Abbreviation: ROC, receiver operating characteristic. AUC, area under the curve.

We further evaluated the prognosis prediction performance of significant prognosis-related genes (*p* < 0.05 in Cox analysis) in metastatic BRCA by ROC analysis. The ROC curve indicated that AGR2 presented the best prediction performance for the survival probability of BRCA metastatic samples in the GSE124647 dataset (Figure 8A). In the GSE175692 dataset, the prognostic performance of AGR2 was next only to ESR1 and TFF1 (Figure 8B). These results suggested the favorable performance of AGR2 for predicting prognosis in BRCA metastasis.

Also, we conducted a nomogram to assess the contribution of these hub genes (*p* < 0.05 in Cox analysis) on the survival probability of patients with BRCA metastasis using 2 datasets. Among 11 prognosis-related genes in GSE175692, AGR2 made the largest contribution to the survival probability of patients, contributing 100 scores (Figure 8C). Among 4 prognosis-related genes in GSE124647, AGR2 also presented the highest contribution score for overall survival probability of patients as AGR2 contributed 100 points. It followed that AGR2 showed the optimal prognostic performance on the survival probability of BRCA metastatic patients.

Under the combination of Cox regression analysis, Kaplan-Meier analysis, nomogram, and ROC analysis using GSE175692 and GSE124647 datasets, AGR2 was finally identified as the most significant prognosis-related biomarker in BRCA metastasis.

### The Clinical Value of AGR2 in BRCA Bone Metastasis and Primary Breast Tumor

**FIGURE 9:**
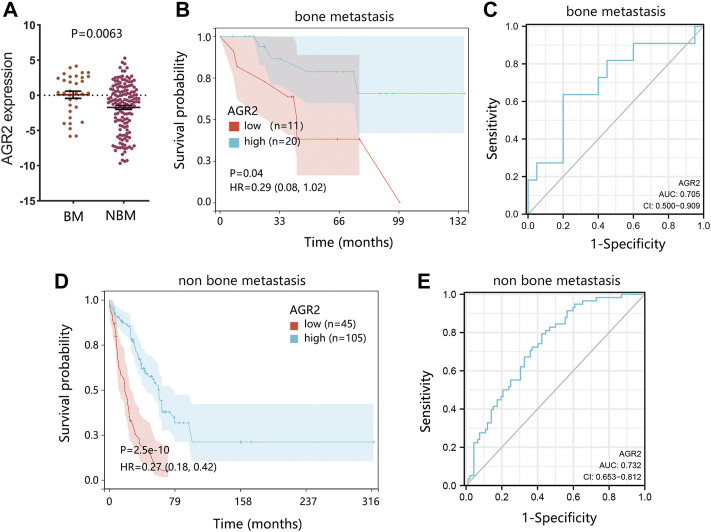
The significance verification of AGR2 in BRCA metastasis using the GSE175692 dataset. **(A)** Differential expression of AGR2 in BRCA samples between bone metastasis and non-bone metastasis groups. **(B)** Correlation between AGR2 mRNA expression and overall survival in BRCA bone metastasis. **(C)** ROC analysis on AGR2 in BRCA bone metastasis. **(D)** Correlation between AGR2 mRNA expression and overall survival in BRCA non-bone metastasis. **(E)** ROC analysis on AGR2 in BRCA non-bone metastasis. For figures **(B)** and **(D)**, the comparison result was presented with *p* value and HR (95% CI). Abbreviation: BM, bone metastasis; NBM, none bone metastasis; HR, hazard ratio; CI, confidence interval; AUC, area under curve; BRCA, breast cancer.

As AGR2 was finally identified as the most significant prognostic biomarker in metastatic BRCA samples, we further explored the detailed role of AGR2 in BRCA bone metastasis using the GSE175692 dataset. Differential expression analysis showed that AGR2 was highly expressed in BRCA bone metastatic cases compared with that in the non-bone metastatic cases (Figure 9A, *p* = 0.0063). AGR2 high expression was conducive to the overall survival of BRCA bone metastatic samples (Figure 9B, *p* = 0.04), and ROC analysis showed that AGR2 presented a favorable performance for predicting a patient’s prognosis (Figure 9C). Also, AGR2 showed significant prognostic value (Figure 9D) and good prediction performance (Figure 9E) in BRCA non-bone metastatic patients.

**FIGURE 10:**
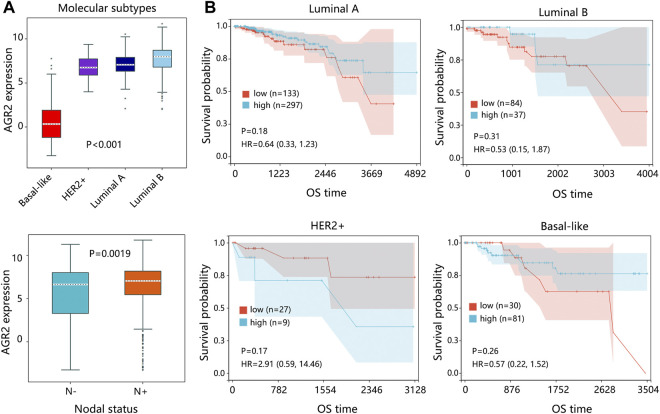
The expression and survival analysis on AGR2 regarding the subtype of the primary breast tumor. **(A)** The expression of AGR2 based on the subtypes of BRCA and nodal status. **(B)** The correlation between AGR2 expression and overall survival time of patients with different subtypes of BRCA. The comparison result was presented with *p* value and HR (95% CI). Abbreviation: HER2, human epidermal growth factor receptor 2; HR, hazard ratio; CI, confidence interval; OS, overall survival; BRCA, breast cancer.

Due to the significance of molecular subtypes of BRCA, we also explored the expression pattern and prognostic impact of AGR2 in primary breast tumors. The results indicated that AGR2 expression was related to the subtypes and nodal status of the primary tumor (Figure 10A). Kaplan-Meier analysis showed that expression of AGR2 was not related to the prognosis of patients under different subtypes (Figure 10B).

### AGR2 Associated Mechanism Exploration in Metastatic BRCA

**FIGURE 11:**
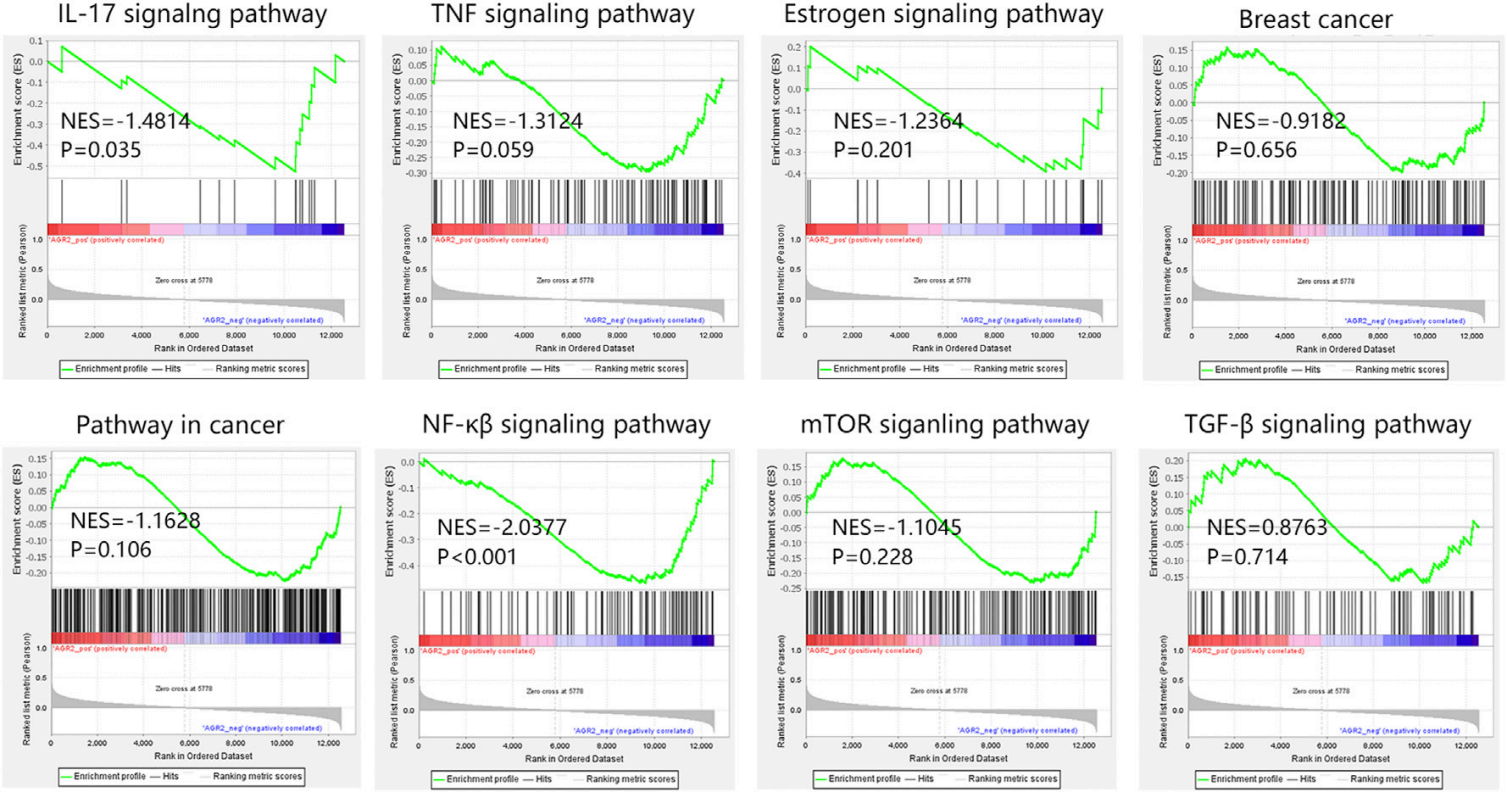
The GSEA analysis on AGR2 in BRCA metastatic samples in GSE124647. Abbreviation: BRCA, breast cancer; GSEA, gene set enrichment analysis; NES, normalized enrichment score; AGR2, Anterior Gradient 2; IL-17, interleukin-17; TNF, tumor necrosis factor; NF-κβ, nuclear factor kappa-β; mTOR, mammalian target of rapamycin; TGF-β, transforming growth factor β.

Due to the importance of AGR2 in BRCA metastatic samples, AGR2-associated pathways were also explored through GSEA analysis. According to our KEGG analysis, 15 hub genes were involved in the cancer pathway, IL-17 signaling pathway, TNF signaling pathway, breast cancer, and estrogen signaling pathway. Hence, we further explored the relationship between AGR2 and these five pathways in metastatic BRCA samples (Figure 11). Our results showed that AGR2 was negatively related to these five pathways, but only the IL-17 signaling pathway showed statistical significance (NES = −1.4814, *p* = 0.035). In addition, we also explored the correlation between AGR2 and three significant metastasis pathways. The result showed that AGR2 negatively correlated with the NF-κβ signaling pathway (NES = −2.0377, *p* < 0.001), but did not relate to mTOR and TGF-β pathways. Briefly, AGR2 might significantly correlate with IL-17 and NF-κβ signaling pathways in metastatic BRCA.

## Discussion

In this study, we detected 74 BRCA bone metastasis-related DEGs by bioinformatics analysis. Among the whole PPI network on DEGs, we determined the most significant module which contained 15 hub genes. Then, five significant KEGG pathways associated with 15 genes were detected. Especially, 15 hub genes were mainly related to the IL-17 signaling pathway, referring to the immune system. IL-17 family has been proved to play an important role in the specific organ metastasis of BRCA. Several studies have shown that IL-17A can lead to metastasis to the lungs and bones (19, 20). IL-17E was proposed to be related to lung metastasis formation in BRCA (21). Detailed IL-17 signaling in this study needs further confirmation. Our study also found the involvement of TNF and estrogen signaling pathways. The study has found that CD137, a member of the TNF receptor superfamily, can promote bone metastasis of BRCA by enhancing the migration and osteoclast differentiation of monocytes/macrophages (22). Cletus et al. found that compared to ER-positive BRCA, ER-negative BRCA was more likely to metastasize to the bone but less likely to the brain, liver, and lung, and less likely to result in multiple metastases (23). These pathways are all significantly correlated with metastatic patterns of BRCA.

Furthermore, we gradually conducted univariate Cox analysis, Kaplan-Meier, nomogram, and ROC analyses on 15 genes using two datasets at the same time to identify the most significant prognosis-related gene. It should be noted that a similar study by Yu et al. recently published also revealed the bone metastasis-related DEGs using the same datasets (24), but the overall arrangement of the two reports was different. The Yu et al. study firstly identified the DEGs and evaluated the clinical value of hub genes. Subsequently, they verified vital genes using the other dataset. Our work aimed to filter important hub genes using two datasets at the same time, and finally determined the hub genes according to the comprehensive result of the two datasets. Under the series of filtration, AGR2 was finally determined as the most significant prognosis-related biomarker in metastatic BRCA in this study. AGR2 is a developmentally regulated gene belonging to the protein disulfide isomerase (PDI) gene family (25). A previous study enrolled 107 primary breast tumors of patients who had developed distant metastasis and classified all patients into two groups according to the site of relapse (bone vs. non-bone), finding that AGR2 was highly expressed in the samples with relapse to bone (26). Guo et al. found that AGR2 expression was significantly associated with histologic subtype, histological grade, estrogen status, progesterone status, and its prognostic impact was related to histological grade (27). In this study, we confirmed that the expression and prognostic value of AGR2 were correlated with the subtypes of primary breast tumors. In addition, AGR2 has been identified as a clinically relevant factor that modulated the behavior and response of hormone-dependent cancers such as BRCA and prostate cancer (28). In BRCA, AGR2 expression was related to ER-positive tumors, and AGR2 was directly targeted by ER-alpha, which was preferentially bound in tumors with poor outcomes (29). Lacambra et al. also indicated that the prognostic impact of AGR2 expression could be related to treatment outcome in ER-positive BRCA (30). It followed that the prognostic impact of AGR2 was significantly related to ER status involved in BRCA.

In addition, we found a correlation between AGR2 higher expression and longer overall survival time in BRCA bone metastatic samples. We further explored the potential mechanism associated with AGR2 in metastatic BRCA. Through analyzing the correlation between AGR2 and five KEGG pathways, we found that AGR2 only significantly correlated with the IL-17 signaling pathway with a negative correlation. We speculated that AGR2 high expression was conducive to the prognosis of patients, which might be due to the negative regulation of the IL-17 pathway. The result further confirmed the importance of the IL-17 signaling pathway in metastatic BRCA. At present, regulation of the IL-17 pathway associated with AGR2 in BRCA was not reported. In addition, we also confirmed the relation between AGR2 and three important metastatic pathways. We found that AGR2 was negatively related to the NF-κβ signaling pathway involved in metastatic BRCA. Aberrant activation of the NF-κβ signaling cascade was connected to carcinogenesis (31). The NF-κβ pathway was significantly related to the bone metastasis in BRCA (32). The AGR2 showed a negative correlation with this pathway in our study, suggesting that AGR2 high expression may inhibit the carcinogenesis of this pathway and thus improve the patient’s prognosis.

This study identified an important bone metastasis-related biomarker which correlated with the patient’s prognosis. However, several limitations of this study needed to be acknowledged. The sample size for prognosis analysis in BRCA bone metastasis was insufficient, hence, a larger sample size is needed to enhance the credibility of these results in future works. In addition, pathways associated with AGR2 including IL-17 and NF-κβ signaling pathways were initially identified in this study, and the detailed regulation in BRCA bone metastasis should be verified by functional analysis.

## Conclusion

This study identified 74 DEGs associated with BRCA bone metastasis. A main module in the PPI network among DEGs was determined, which included 15 DEGs. KEGG analysis showed that 15 genes were mainly involved in the IL-17 signaling pathway referring to the immune system. Further, we gradually performed the univariate Cox analysis, Kaplan-Meier, nomogram, and ROC analyses on 15 genes. AGR2 was finally proved to present the most important role, as it showed the largest contribution and optimal prognostic performance on survival probability of metastatic BRCA patients, as well as significant prognostic impact in single bone metastatic BRCA. Pathway analysis found that AGR2 negatively correlated with NF-κβ and IL-17 signaling pathways. The present study provides a useful prognostic biomarker in BRCA bone metastasis, and detailed function in cancer progression needs more functional verification.

## Data Availability

The datasets used and/or analyzed during the current study are available in the following links: https://www.jianguoyun.com/p/DXnjM64Qy_3mChj_-80EIAA.

## Abbreviations

BRCA, breast cancer; DEGs, differentially expressed genes; PPI, protein-protein interaction; ROC, receiver operating characteristic; GSEA, gene set enrichment analysis; AGR2, Anterior Gradient 2; IL, Interleukin; NF-kβ, nuclear factor kappa β.
